# C911: A Bench-Level Control for Sequence Specific siRNA Off-Target Effects

**DOI:** 10.1371/journal.pone.0051942

**Published:** 2012-12-14

**Authors:** Eugen Buehler, Yu-Chi Chen, Scott Martin

**Affiliations:** National Center for Advancing Translational Sciences, National Institutes of Health, Bethesda, Maryland, United States of America; Niels Bohr Institute, Denmark

## Abstract

Small interfering RNAs (siRNAs) have become a ubiquitous experimental tool for down-regulating mRNAs. Unfortunately, off-target effects are a significant source of false positives in siRNA experiments and an effective control for them has not previously been identified. We introduce two methods of mismatched siRNA design for negative controls based on changing bases in the middle of the siRNA to their complement bases. To test these controls, a test set of 20 highly active siRNAs (10 true positives and 10 false positives) was identified from a genome-wide screen performed in a cell-line expressing a simple, constitutively expressed luciferase reporter. Three controls were then synthesized for each of these 20 siRNAs, the first two using the proposed mismatch design methods and the third being a simple random permutation of the sequence (scrambled siRNA). When tested in the original assay, the scrambled siRNAs showed significantly reduced activity in comparison to the original siRNAs, regardless of whether they had been identified as true or false positives, indicating that they have little utility as experimental controls. In contrast, one of the proposed mismatch design methods, dubbed C911 because bases 9 through 11 of the siRNA are replaced with their complement, was able to completely distinguish between the two groups. False positives due to off-target effects maintained most of their activity when the C911 mismatch control was tested, whereas true positives whose phenotype was due to on-target effects lost most or all of their activity when the C911 mismatch was tested. The ability of control siRNAs to distinguish between true and false positives, if widely adopted, could reduce erroneous results being reported in the literature and save research dollars spent on expensive follow-up experiments.

## Introduction

Initially a bench-level technique for targeting single genes for down-regulation, siRNAs have grown into a major source of high-throughput data with functional screens that attempt to access the involvement of the entire transcriptome in a particular biological process using tens of thousands of siRNAs [1]. Low validation rates and the lack of overlap between genes identified in different screens targeting the same pathway [2] has led to a increased understanding of the prevalence and mechanisms of siRNA off-target effects [3]. Recent research has leveraged analysis of seed sequences in siRNA screens to identify likely false positives due to off-target effects [4] and infer transcripts responsible for off-target phenotypes [5], [6], but these methods rely on the statistical analysis of large sets of data and are not applicable to smaller screens and bench-level experiments using a small number of siRNAs.

From the beginning of siRNA use as an experimental method, concern has existed about false positives due to lack of specificity [7], [8]. Although it has been previously noted that scrambled siRNAs are probably a sub-optimal control, a validated alternative has not been available. Standard non-silencing controls can be used to control for general effects common to transfection with any siRNA, but they cannot control for off-target effects specific to a given siRNA, which are determined by the seed sequence (bases 2–8 at the 5′ end of the siRNA strand loaded into RISC) [9] and will thus vary from siRNA to siRNA.

**Figure 1:**
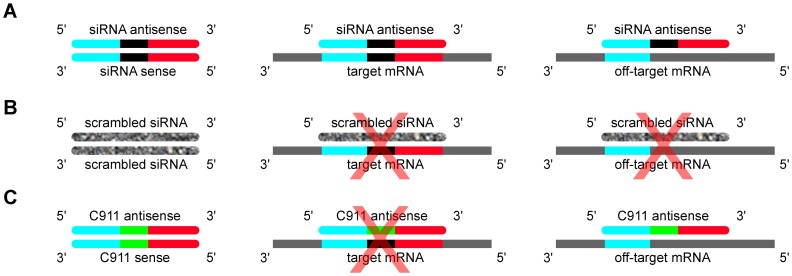
On and off-target effects of siRNAs and their controls. An siRNA (**A, left panel**) consisting of two complementary 19-mers of RNA (with two-base overhangs) is divided here conceptually into the 5′ end of the anti-sense strand (teal) the middle of the siRNA (black) and the 3′ end of the anti-sense strand (red). siRNAs are designed to be the reverse-completment of the mRNA sequence they are targeted to down-regulate (**A**, **middle panel**), but matches of the seed sequence of an siRNA to the 3′UTR of other mRNAs can result in their off-target down-regulation as well (**A**, **right panel**). A scrambled siRNA (**B**) eliminates the match to the target mRNA and thus will not down-regulate it, but also eliminates the off-target effects due to matches to the seed sequence (while, perhaps, creating new off-target effects against the new seed sequence). The C911 mismatch control (**C**) reduces or eliminates the down-regulation of the targeted mRNA by taking the complement of the middle three bases (green), but maintains the off-target effects of the original siRNA by keeping anti-sense and sense strand seed sequences intact. In this manner, comparison of effects elicited by the original siRNA and the C911 mismatch control should allow us to distinguish phenotypes that are due to down-regulation of the intended target rather than off-target effects.

To find a suitable control for individual siRNAs, a modification is required that will eliminate on-target effects while retaining the same off-target effects. We propose that this can be accomplished by maintaining guide and passenger strand seed sequences of the siRNA (bases 2–8 and bases 12–17 respectively) and each of their respective efficiencies loading into the RISC complex, which is probably determined in part by the GC-asymmetry between the terminal bases on either end of the siRNA (bases 1–3 and 16–19) [10]. We test two mismatch designs that meet these requirements: C10, which is the same siRNA except that base 10 is the complement of the original siRNA, and C911, which is the same siRNA except that bases 9 through 11 are the complement of the original siRNA (**Figure 1**). Previous work has determined that mismatches at base 10 of an siRNA could effectively differentiate between mRNAs that differ by a single base [11].

It is worth noting that the idea of using a centrally mismatched siRNA as an experimental control is not a new one. Bryan Cullen proposed in 2006 [12] that “one could also test an siRNA or an shRNA mutant bearing a central ≥3-nt mismatch to the target mRNA that leaves the seed region unchanged”. However, our review of the literature does not reveal any systematic test of this idea that would allow researchers to employ mismatch siRNAs as a control with confidence that it will allow them to distinguish between true and false positives.

## Materials and Methods

Testing the efficacy of the C10 and C911 mismatch control designs required us to identify a gold standard set of true and false positive siRNAs. To find siRNAs which have a significant inhibitory effect on a constitutively expressed reporter luciferase, a previously performed whole genome screen, briefly described below, was analyzed.

HEK293 cells harboring CMV-driven firefly luciferase were obtained from Promega and cultured in DMEM, 10% FBS. Cells were passaged every 3 days. Screening was conducted using the Ambion Silencer Select Human Genome siRNA Library Version 4. This collection targets ≈21,500 genes, with the vast majority of genes targeted by 3 independent, non-pooled siRNAs. For screening, siRNA reagent (0.8 pmol) was spotted into white solid bottom 384-well plates (Corning 3570) using a VPrep liquid handler (Velocity11, Agilent Technologies) integrated into a BioCel robotic platform (Agilent Technologies). All screening plates had a full column (16 wells) of both negative (Ambion SilencerSelect Negative Control #2) and positive control (PLK1 Ambion SilencerSelect siRNA, cat# s448). Positive control served to assess transfection efficiency and assay performance, whereas the median value of each plate’s negative control column was used as a method to normalize corresponding sample wells. Lipofectamine RNAiMax (Invitrogen, 0.07 µL) was added to plate wells in 20 µL of serum free media (DMEM) using a WellMate dispenser (Thermo Scientific). Transfection reagent and siRNA were complexed for 45 minutes at ambient temperature before adding cells (1000) in 20 µL of media containing 20% serum (WellMate, Thermo Scientific). This yielded final transfection mixtures comprising 20 nM siRNA in media containing 10% serum (standard for the growth of HEK293 cells). The cells were then cultured for 72 hours at 37°C in 5% CO_2_ prior to addition of OneGlo luciferase assay reagent (Promega). Luminescence was measured using an EnVision Multilabel Plate Reader (PerkinElmer).

It should be noted that the median assay response (81.8%) in the screen was well below the negative control (100%, by definition) used to normalize the screen results and subsequent assay results. This may be due to off-target effects of the negative control. Alternatively, the median assay response could be lower than the negative control because there are many more mRNAs that when down-regulated would interfere with transcription as compared to mRNAs that when down-regulated would promote or increase transcription of the reporter. To aid in interpretation of the results, the median assay response from the screen is indicated on the graphs of experimental results.

**Figure 2:**
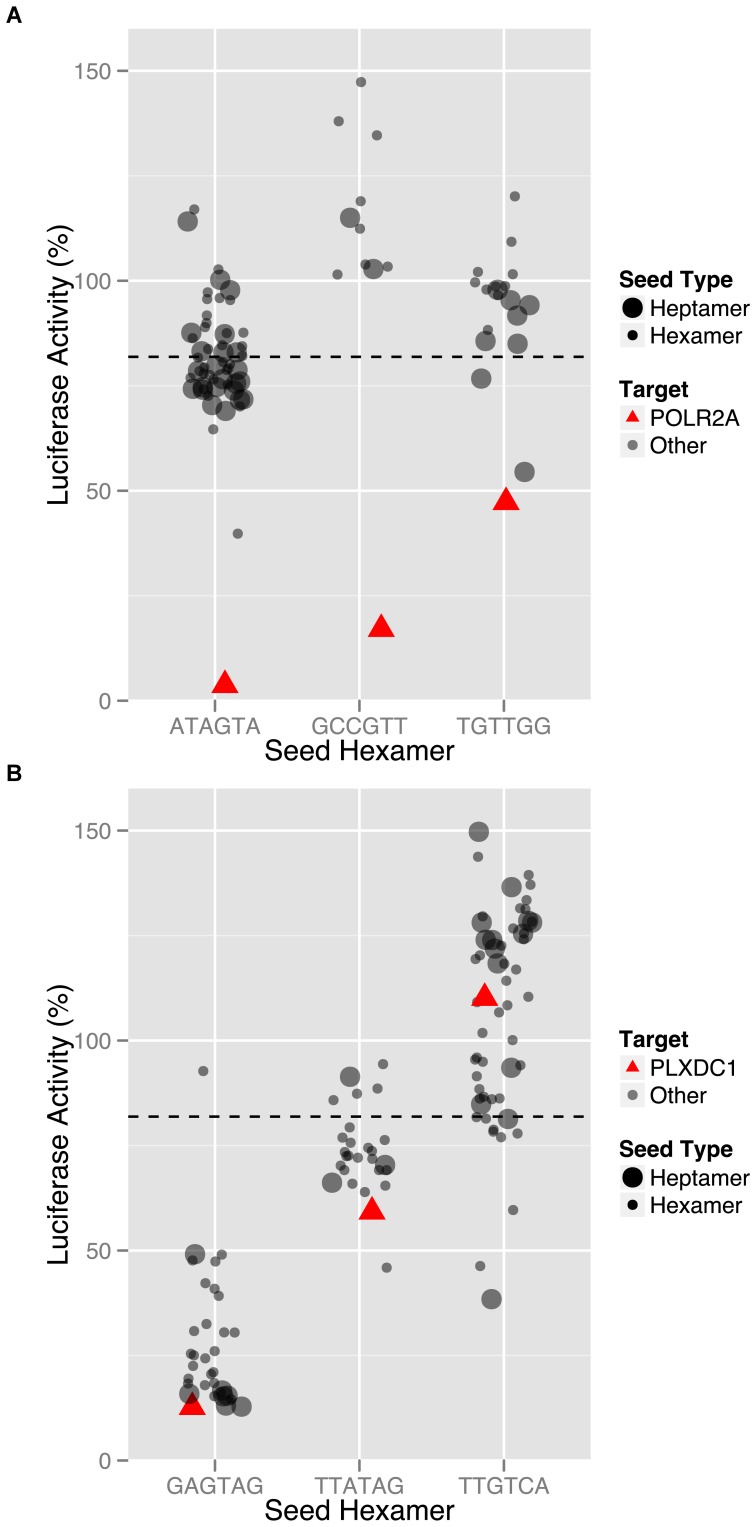
Examples selected for test set. Common seed analysis plots for two genes which had an siRNA showing significant knockdown (greater than 2-fold decrease in luciferase signal in comparison to non-silencing control). The dashed line represents the median response for the whole genome library. (**A**) POLR2A was selected as a true positive because multiple siRNAs generated the same phenotype, the role of RNA polymerase in transcription is already established, and siRNAs containing the same seed sequences (grey circles) did not show a trend towards inhibition of the reporter. (**B**) In contrast, PLXDC1 was selected as a false positive because it has no known role in transcription, other siRNAs against the same gene fail to elicit the same phenotype, and there is an obvious trend of down-regulating the reporter for all other siRNAs containing the same hexamer (GAGTAG) or heptamer seed sequence.

From the siRNAs in this screen that significantly down-regulated the luciferase reporter (more than 2-fold inhibition), a gold standard set of ten true positives and ten false positives was selected (**Figure 2** **,** **Table 1**, **and Data S1**). True positives were selected based on their having a reasonable biological connection to transcription, multiple siRNAs designed against the same mRNA having the same approximate phenotype, and little or no evidence that the phenotype was due to seed-based off-target effects (based on the Common Seed Analysis [4] plot). Conversely, false positive siRNAs were selected based on the targeted gene having no known function in transcription, little or no activity in the assay from other siRNAs designed against the same mRNA, and clear evidence of seed-based off-target effects. False positives were further restricted to have unique seed sequences to ensure diversity of the gold standard set.

**Table 1:** siRNAs Selected as True and False Positives.

Gene Symbol* ofSelected siRNA	Luciferase Activity(Percent ofnegative control)for SelectedsiRNA	Luciferase Activity(Percent ofnegative control)for siRNA #2Against Gene	Luciferase Activity(Percent ofnegative control)for siRNA #3Against Gene	Category (True Positive or False Positive)	Median Luciferase Activity of all siRNAs having the same Seed Sequence as the Selected siRNA	StatisticallySignificant Bias ofsiRNAs Havingthe Same SeedSequence
PLXDC1	12.7	59.2	110.1	FP	15.4	TRUE
hCG_1989844	13.6	26.0	92.2	FP	25.2	TRUE
PPAP2C	14.6	53.1	83.6	FP	59.5	FALSE
C4orf21	16.6	73.4	73.8	FP	39.1	TRUE
LHX1	17.5	96.1	99.4	FP	33.9	TRUE
B3GNT7	19.4	77.2	102.5	FP	40.7	TRUE
hCG_2045830	19.8	71.2	NA	FP	34.9	TRUE
ZFP36L2	19.8	74.3	95.9	FP	31.1	TRUE
LOC646570	23.8	44.3	82.4	FP	45.3	TRUE
LOC645504	24.4	48.8	93.1	FP	35.4	TRUE
POLR2A	3.7	17.1	47.3	TP	76.3	FALSE
POLR2B	13.8	18.3	55.9	TP	73.5	FALSE
PCF11	14.6	18.9	34.7	TP	103.5	FALSE
POLR2J3	17.2	11.8	23.8	TP	77.7	FALSE
POLR2I	17.3	25.2	53.1	TP	84.4	FALSE
RPS27A	17.9	25.4	42.4	TP	65.8	FALSE
SON	20.1	27.4	29.5	TP	58.7	FALSE
RPL36	20.9	27.6	35.8	TP	58.1	FALSE
EIF2S3	22.0	28.7	62.9	TP	71.4	FALSE
POLR2D	23.5	17.2	15.0	TP	79.0	FALSE

* Not all Gene Symbols are HUGO approved. Some siRNAs were designed against genes which were later reclassified as pseudo-genes.

To test the ability of different control siRNAs to distinguish between the selected true positives and false positives, four siRNAs were synthesized for each of the original 20 siRNAs: an unmodified siRNA with the same sequence as the originally identified duplex and with no chemical modifications, a scrambled siRNA with the same base frequency as the original siRNA in a different order (generated by an online tool for scrambled siRNAs, http://www.sirnawizard.com/scrambled.php), the siRNA with base 10 complemented (C10), and the siRNA with bases 9 through 11 complemented (C911). Each of these eighty siRNAs was then tested in the assay for luciferase activity used in the original genome-wide screen, with three replicates per siRNA. Results were normalized to percent of activity observed with a commercially available non-targeting control (Ambion Silencer Select Negative Control 2, Life Technologies™).

The statistical significance of the difference between each of three controls and the original siRNA was calculated using Student’s t-test and p-values of less than or equal to 0.05 were designated as being statistically significant. All statistical analysis was performed in R [13], and figures were generated using ggplot2 [14] and Inkscape (http://inkscape.org/).

## Results

**Figure 3:**
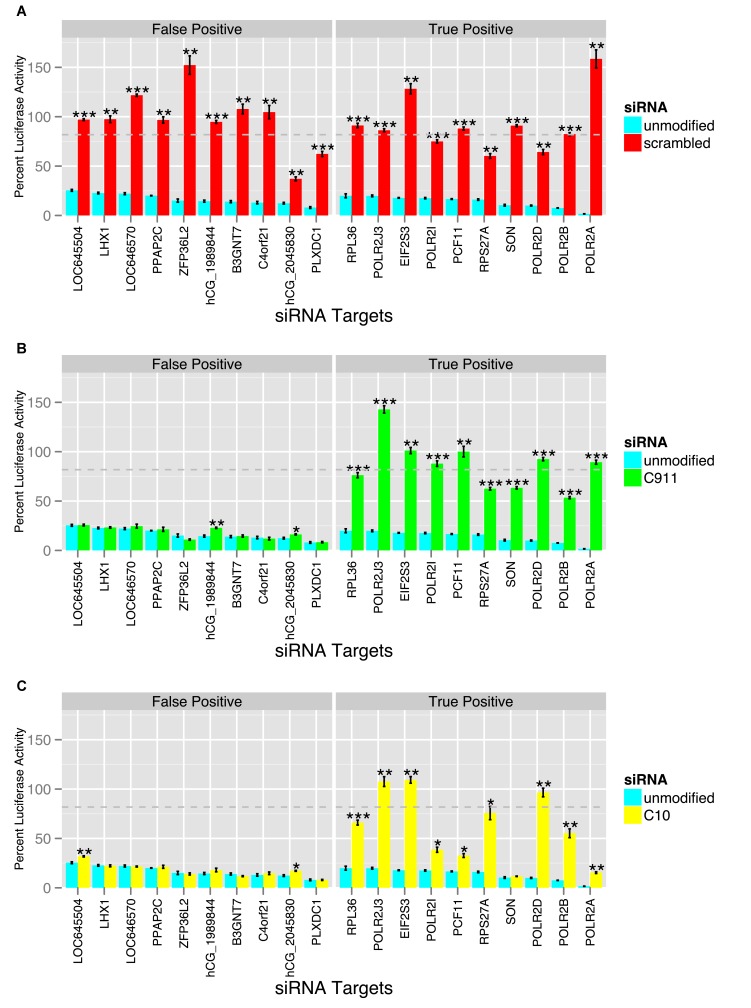
Results for three different classes of negative control. siRNAs which downregulate a luciferase reporter more than two-fold (**teal, panels A,B, and C**) were selected and categorized as either true or false positives based on biological annotation, the activity of other siRNAs against the same gene, and observed activity of other siRNAs with the same seed sequence. Three types of negative control were then tested for their ability to distinguish true positives from false positives. As expected, scrambled versions of the siRNAs (**red, panel A**) reduced or eliminated their activity, regardless of whether it was on (true positive) or off-target (false positive). In contrast, the C911 mismatch design (**green, panel B**) showed a large reduction in activity for only true positive siRNAs, indicating that changing bases 9–11 of the siRNAs to their complement successfully disrupted on-target activity while maintaining off-target activity. The C10 mismatch design (**yellow, panel C**) also maintained off-target effects for false positives, but in some cases failed to reduce the on-target phenotype observed in the true positive group as drastically as the C911 mismatch. Statistically significant p-values for the t-test are marked by *** (p-value < = .001), ** (p-value < = .01), and *(p-value < = .05). The dashed line represents the median response for the whole genome library. Error bars represent the calculated standard error (standard deviation divided by the square root of the number of observations).

As expected, scrambling the siRNA sequences (**Figure 3A**) eliminated most or all of their activity in the assay, regardless of whether they were true or false positives. In contrast, the C911 mismatch siRNAs (**Figure 3B**) showed a large reduction in activity for all of the true positive siRNAs and for none of the false positive siRNAs, demonstrating that the C911 mismatch control effectively reduces on-target effects while maintaining off-target effects, allowing experimental discrimination between true and false positives. For two of the C911 false positives, there was a small but statistically significant difference between the mismatch control and the original siRNA, indicating that the maintenance of the off-target effect by the C911 mismatch may not always be perfect. The C10 mismatch design (**Figure 3C**) also showed ability to distinguish between true and false positives, although the amount of on-target activity reduction was significantly less than in C911 for several siRNAs (SON, POLR2A, PCF11, POLR2I). As with C911, two of the C10 mismatch siRNAs against putative false positives showed small but statistically significant differences in activity from the original siRNAs.

## Discussion

It is possible that there are sequence specific off-target effects that are mediated in part by bases 9 through 11 of the siRNA, although we do not see evidence for that in these experiments. If this were the case, the C911 modification might significantly diminish off-target effects for an siRNA and lead to a false positive. Likewise, it is possible that there are siRNAs for which the on-target effect will not be significantly diminished by the C911 modification, which could lead to a false negative. Since the C911 modification worked for 20 of 20 siRNAs in these experiments, we can infer that the failure rate will be small but we cannot guarantee it will be 0.

Although the siRNAs for this study were chosen to fall into one of two categories (phenotype due to on-target effect or phenotype due to off-target effect), it is possible, even likely, that an siRNA’s observed activity could be a combination of on-target and off-target effects. In this case, we would expect that the C911 control would continue to show activity in the assay, but perhaps less activity than the original siRNA which also had on-target effects. For this reason the correct comparison is to ask if there is a reproducible, statistically significant difference between the C911 control and the unmodified siRNA, not if the C911 control has or does not have activity in the assay.

Finally, it is important to note that failing to disprove the null hypothesis that a gene is not involved in the pathway of interest is not the same as proving the null hypothesis. As has been the case previously, an siRNA against a biologically relevant mRNA may fail to elicit a phenotype for a number of reasons, including knockdown that is insufficient to elicit a phenotype distinguishable from noise in the assay. These caveats will continue to apply when using the C911 control.

In light of the promising initial results observed for our test set, we believe researchers should move away from using scrambled/non-targeting controls for individual siRNA experiments, which we have shown experimentally have no utility in distinguishing between true and false positives and will only lend undeserved confidence to results that may fail to confirm in more time consuming rescue experiments. Instead, the use of the C911 mismatch control has shown excellent ability to detect false positives and can easily be incorporated into experimental designs. Eventually, siRNA vendors could offer siRNAs and their C911 mismatch control for sale together, saving investigators the time and expense of ordering these controls separately. It would also be possible to synthesize entire libraries with their mismatch controls included, which could allow for more accurate siRNA screening, especially for small libraries that are not amenable to larger scale statistical analyses.

## Supporting Information

Data S1 **Experimental Data.** Excel spreadsheet containing sheets for the selected siRNAs, the synthesized sequences, and the experimental results plotted in **Figure 3**.
(XLSX)

Plots S1 **Common Seed Analysis (CSA) Plots for Gold Standard Selections.** This supplemental file contains twenty Common Seed Analysis (CSA) plots, one per page. Each CSA plot is for one of 20 siRNAs chosen as a true or false positive, along with the other siRNAs intended to target the same gene. The dashed line represents the median response for the whole genome library. The y-axis is percent luciferase activity compared to negative control. Each siRNA tested against the gene of interest is plotted in its own column as a red triangle. In the same column, siRNAs tested against different genes/mRNAs that had the same heptamer seed sequence (bases 2–8, large grey circles) or hexamer seed sequence (bases 2–7, small grey circles) are plotted. When all siRNAs with the same seed sequence have roughly the same phenotypic effect as the siRNA of interest, we can conclude that the phenotype is likely due to seed-based off-targeting and is not specific to the intended target.
(PDF)

File S1 **Code for CSA Plots.** Computer code in the R programming language that was used to generate the CSA plots in **Figure 2** and **Plots S1**. Requires the ggplot package.
(R) Click here for additional data file.
