# Supplementary figures and images for: C911: A Bench-Level Control for Sequence Specific siRNA Off-Target Effects
Source: PLoS One. 2012 Dec 14;7(12):e51942. doi: 10.1371/journal.pone.0051942 (PMC3522603; doi:10.1371/journal.pone.0051942)

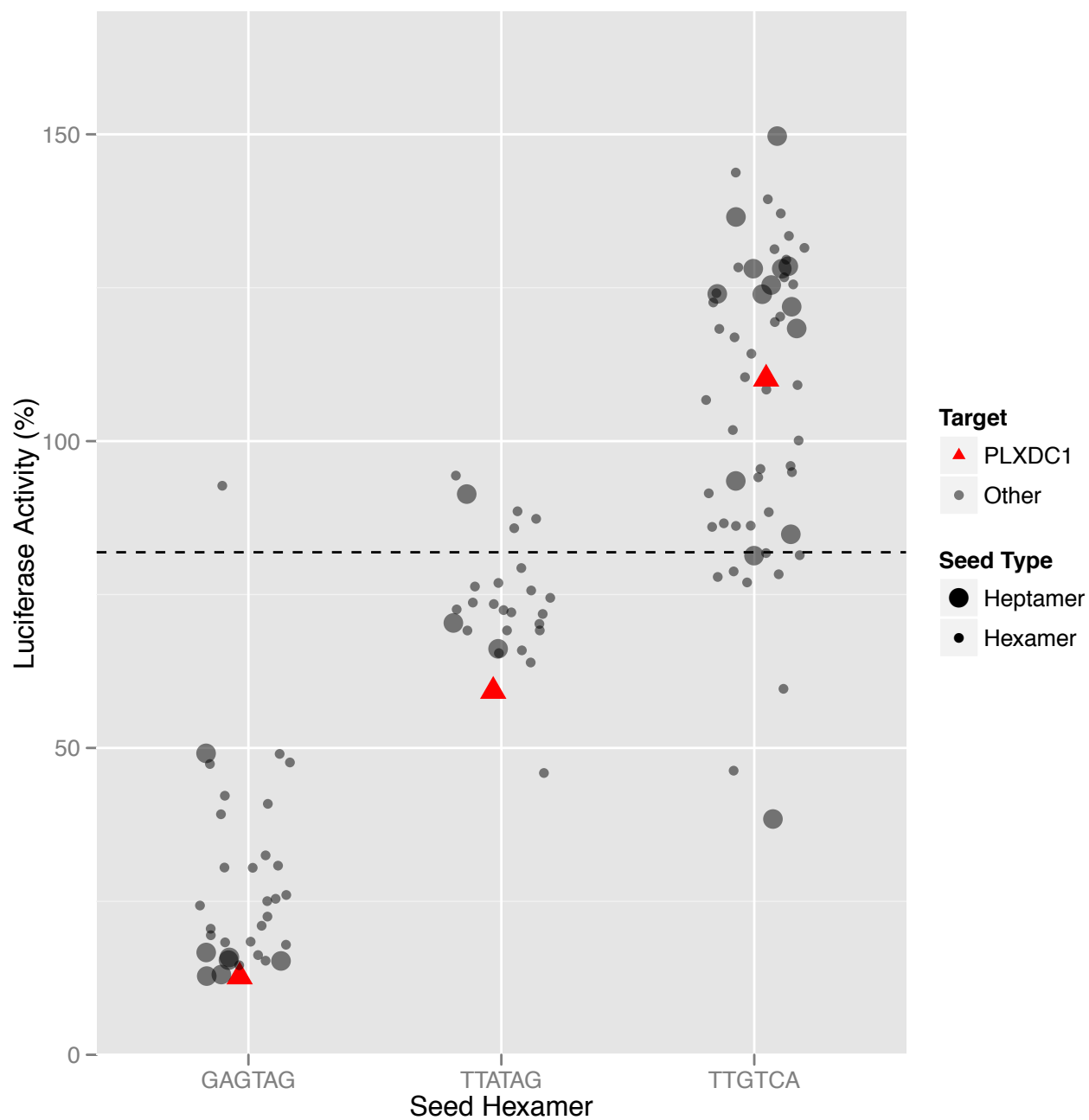

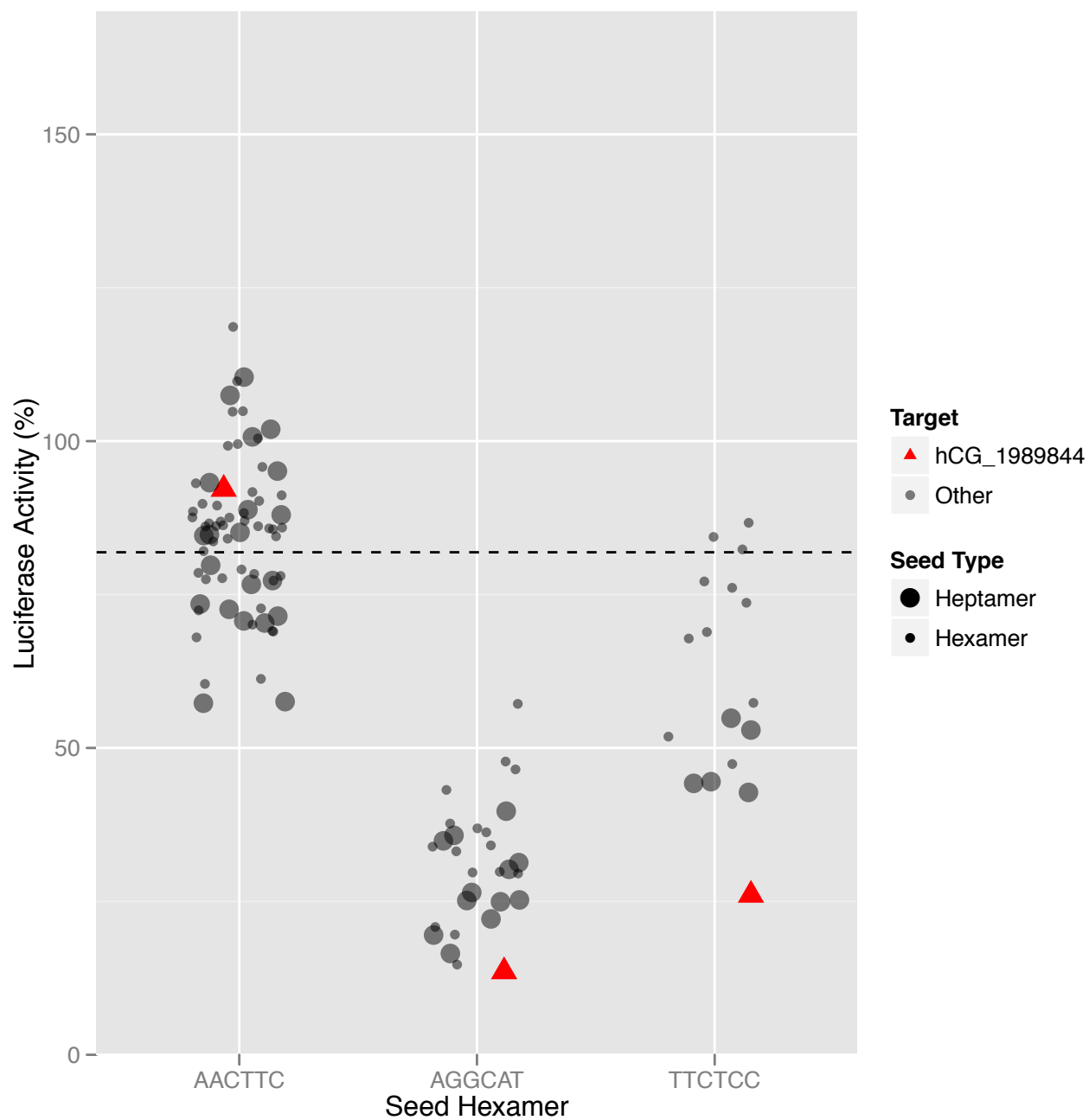

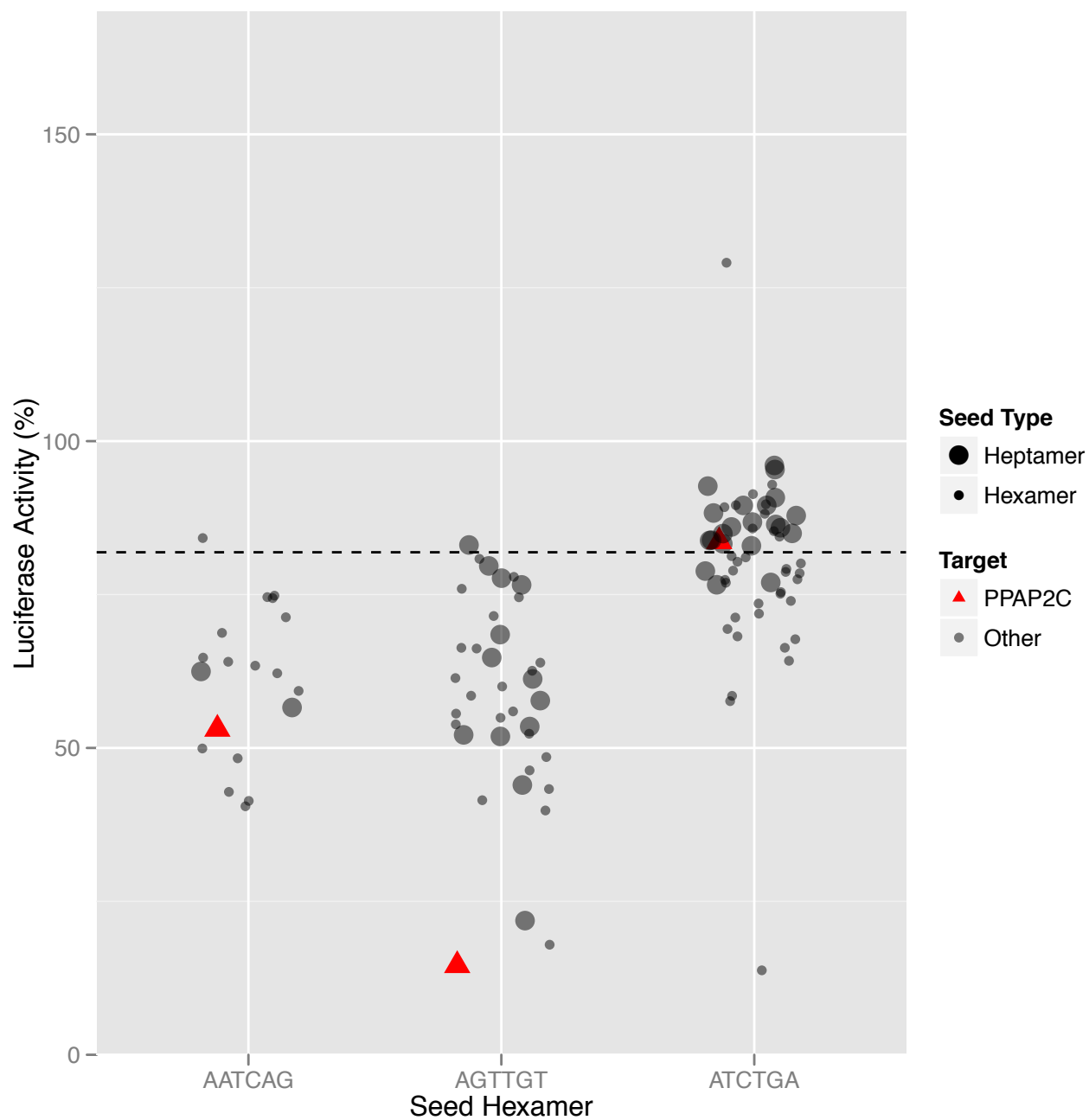

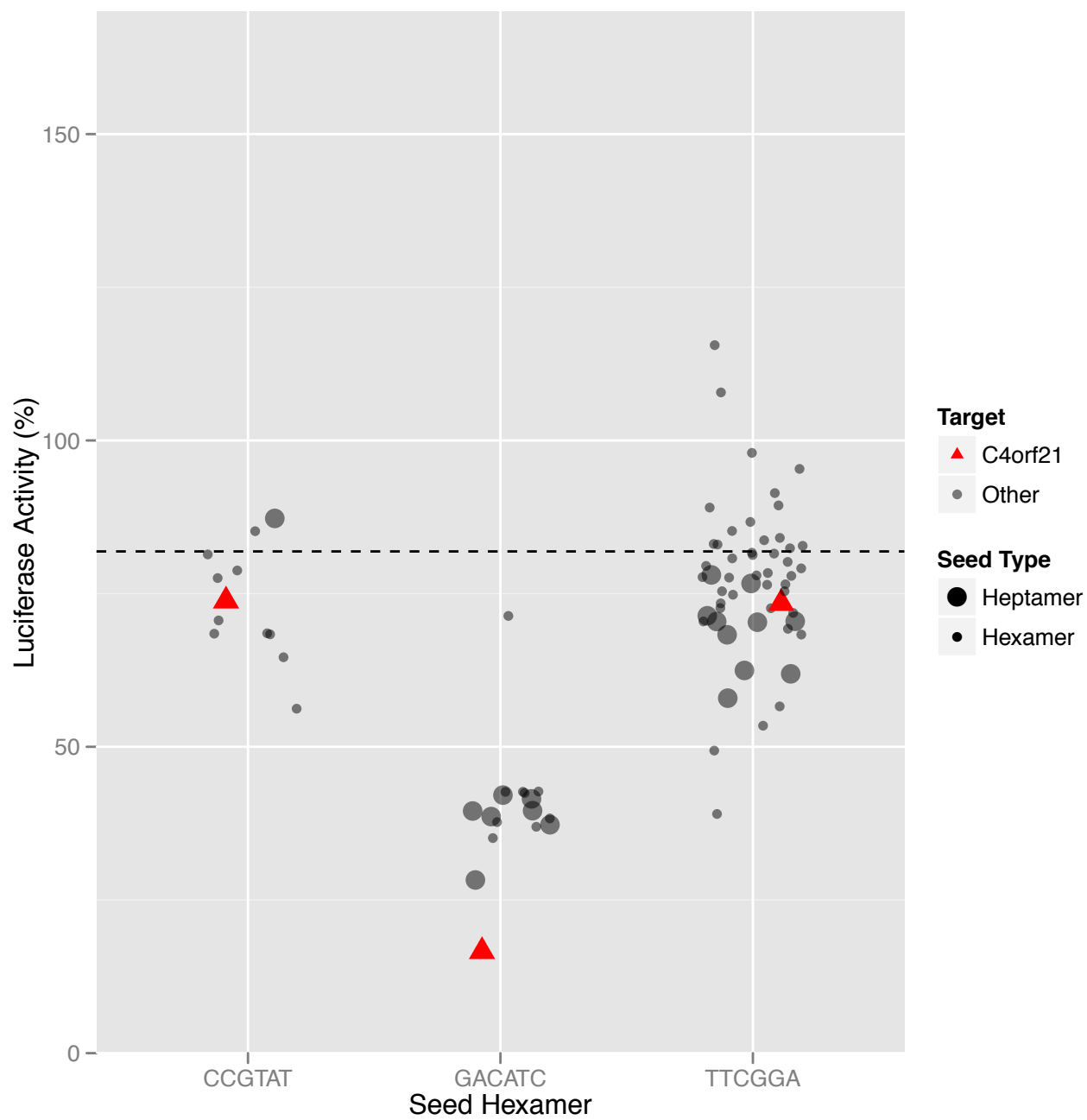

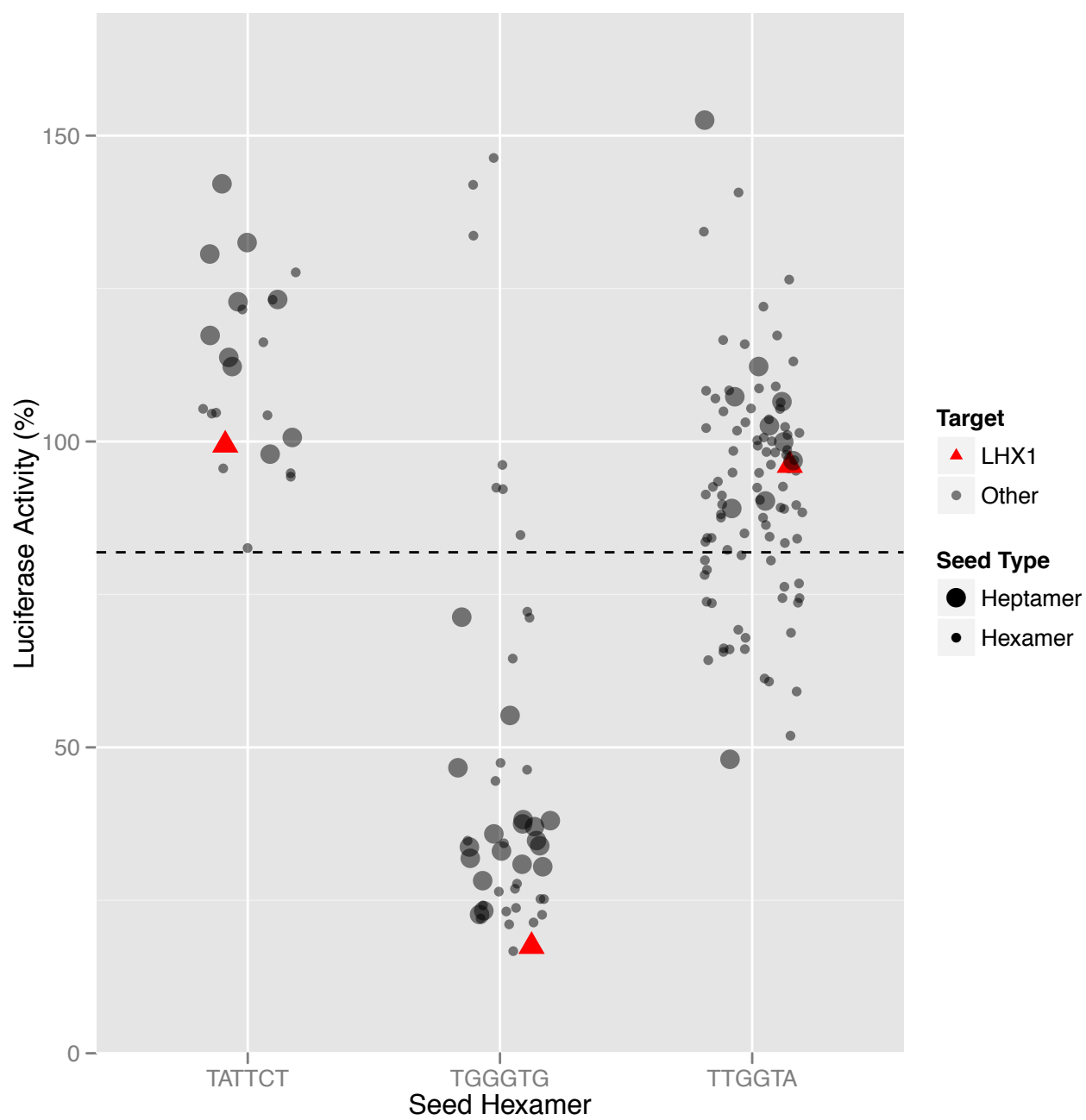

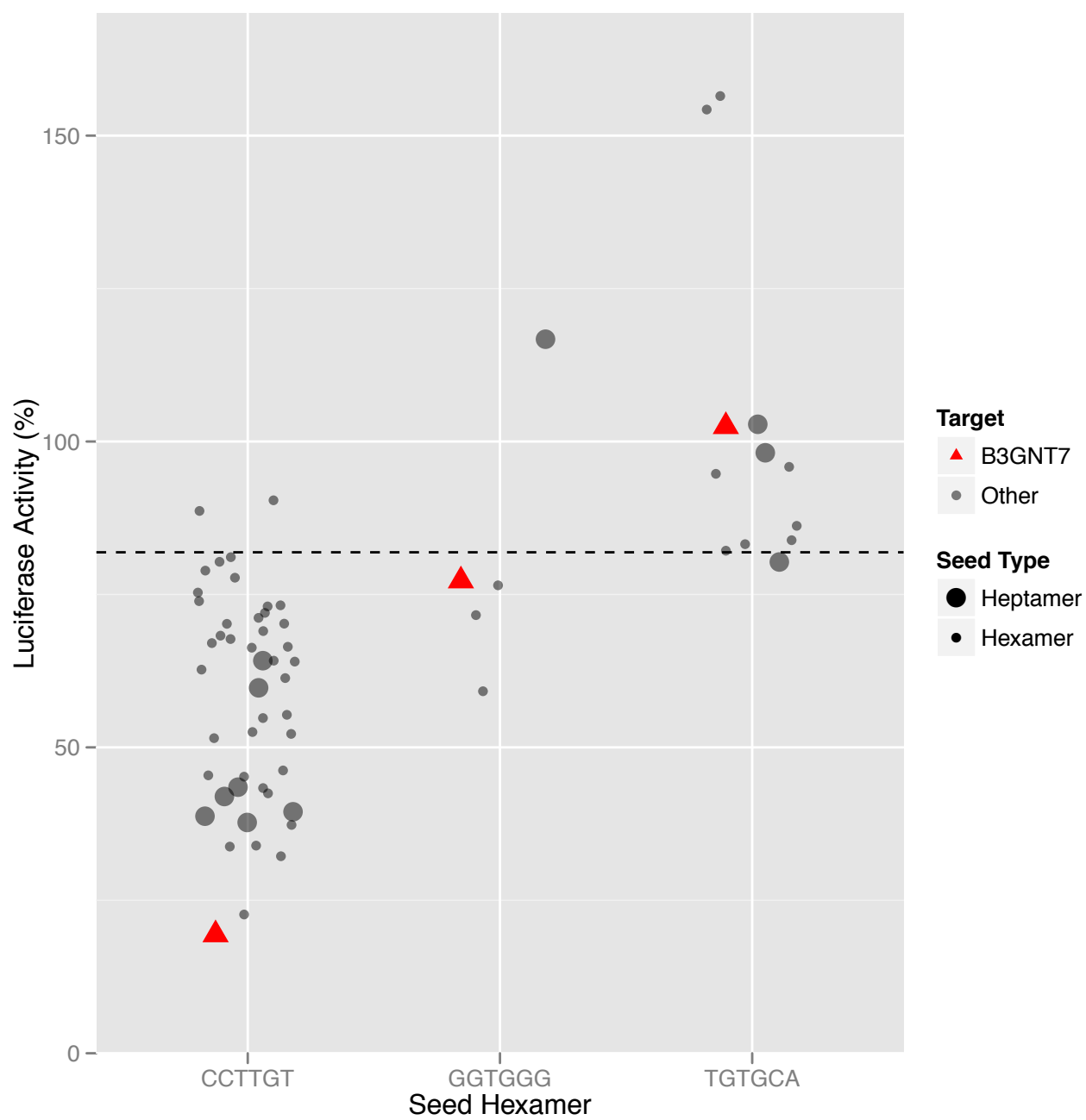

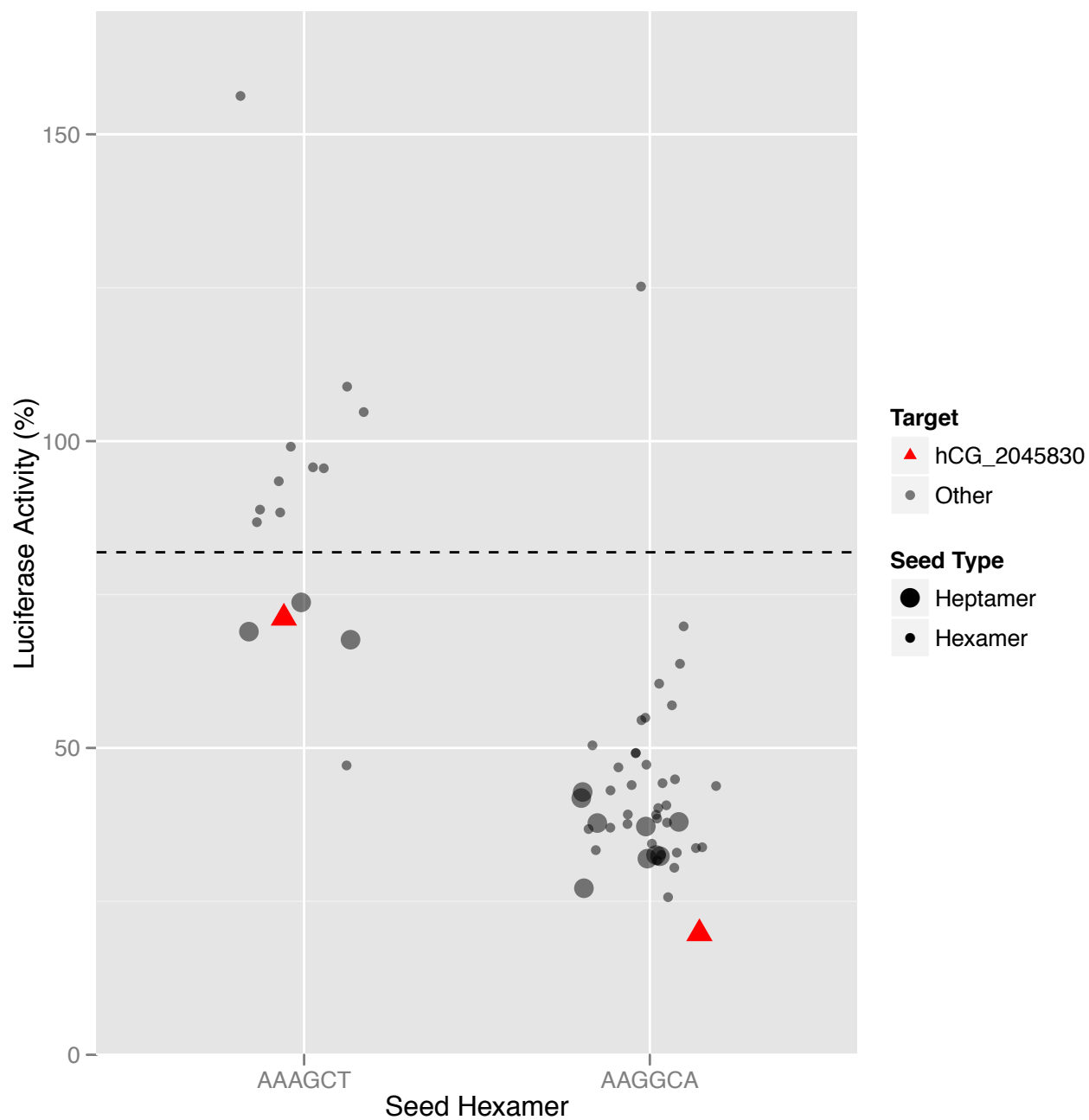

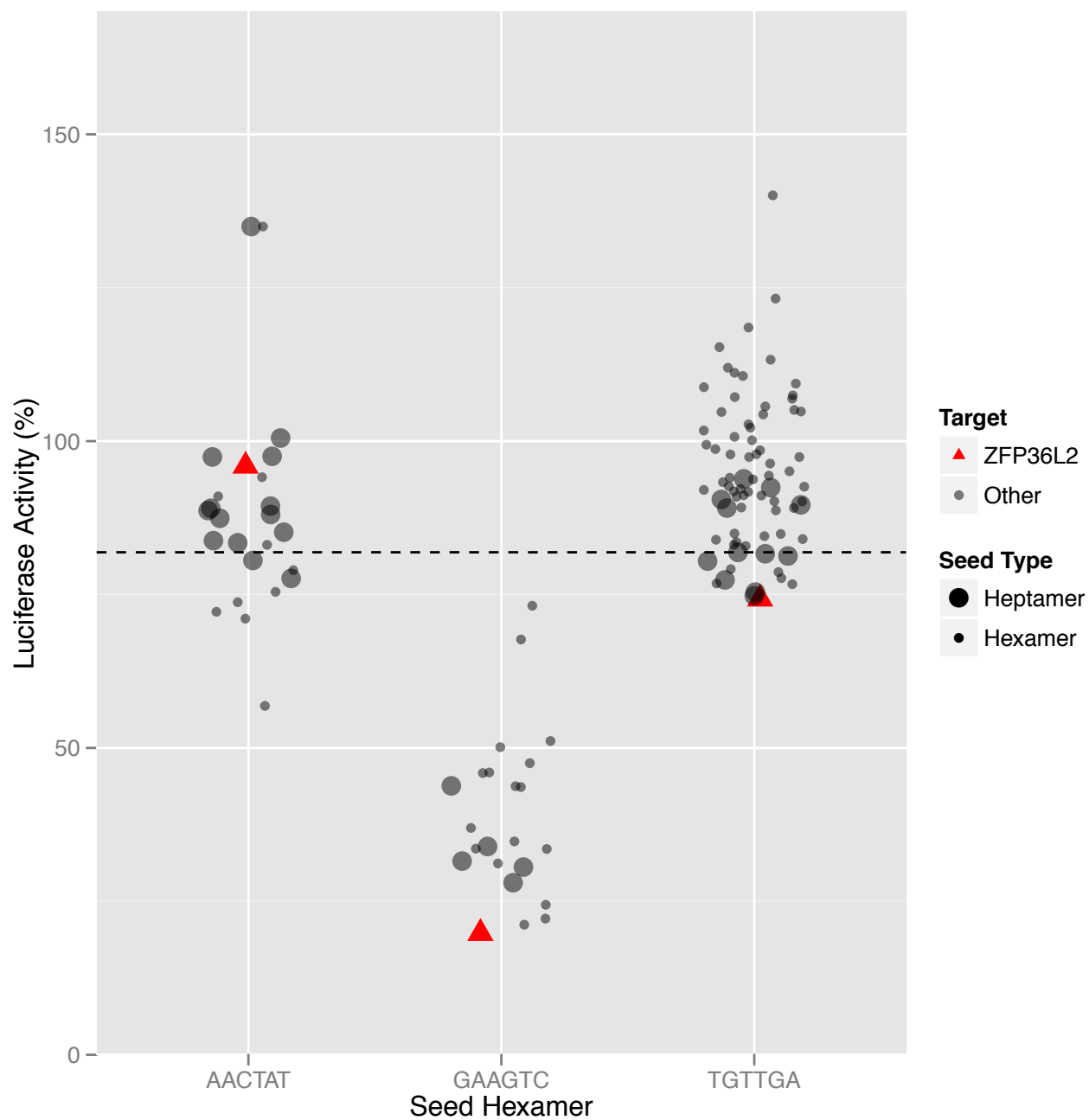

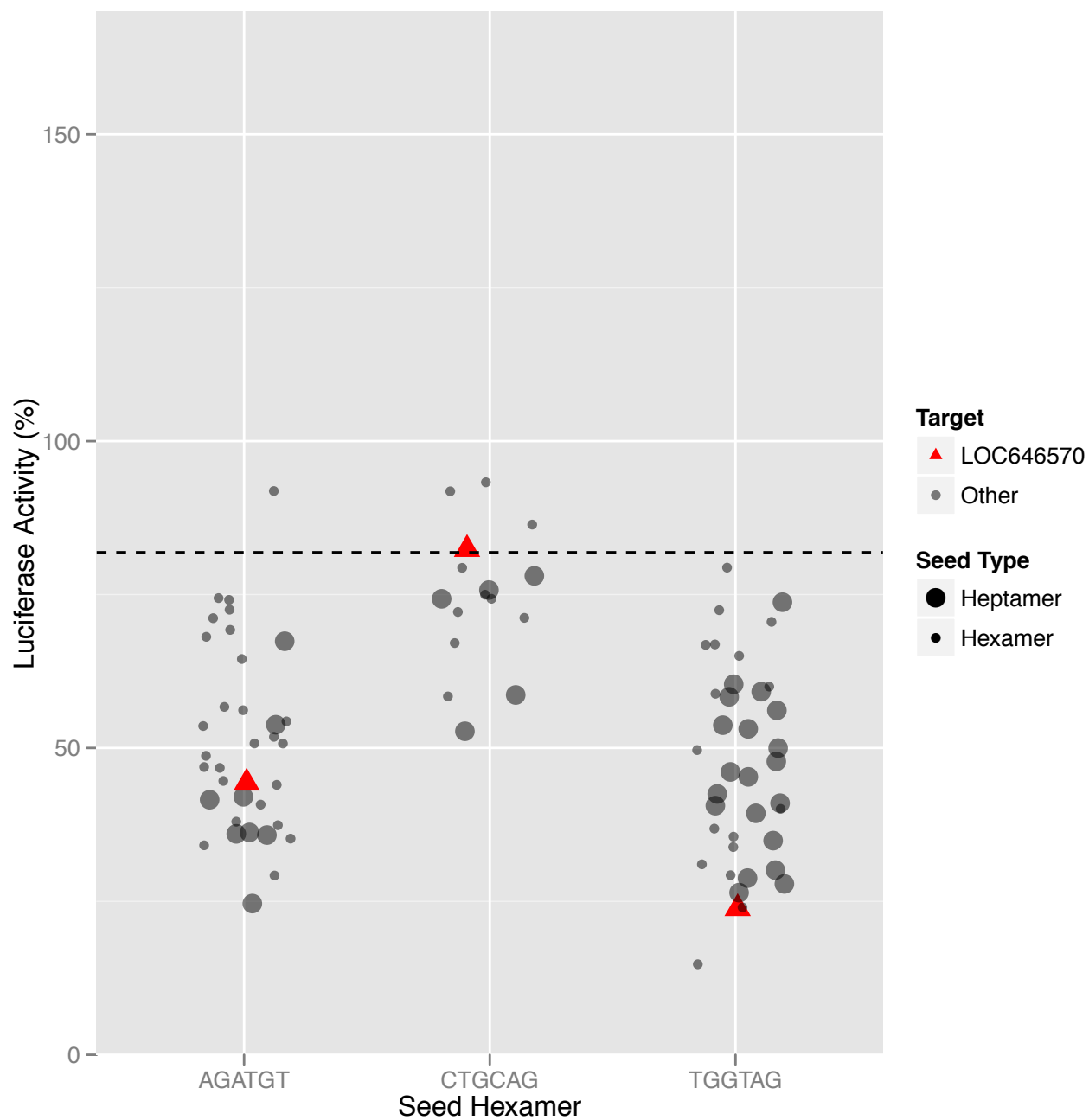

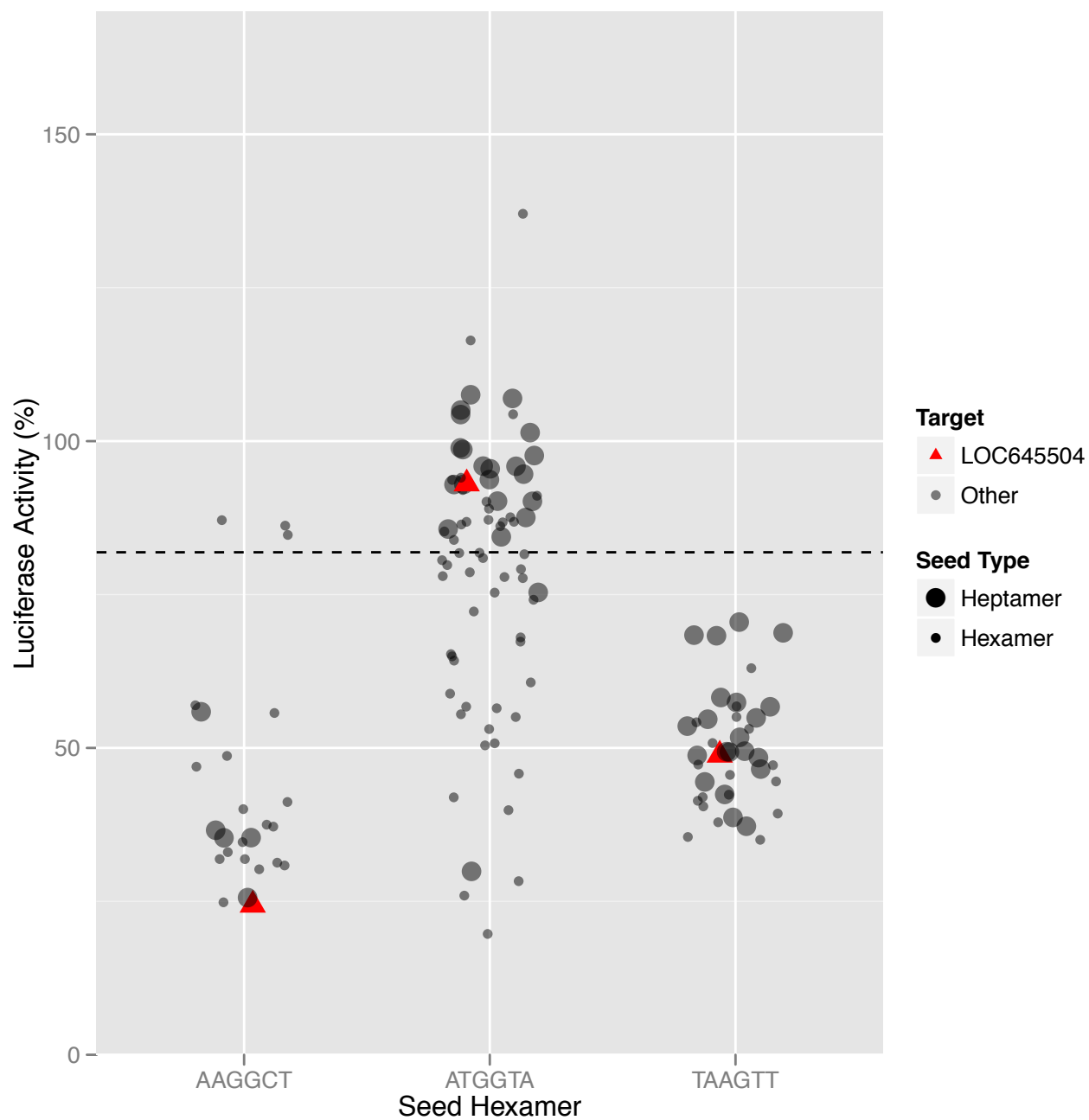

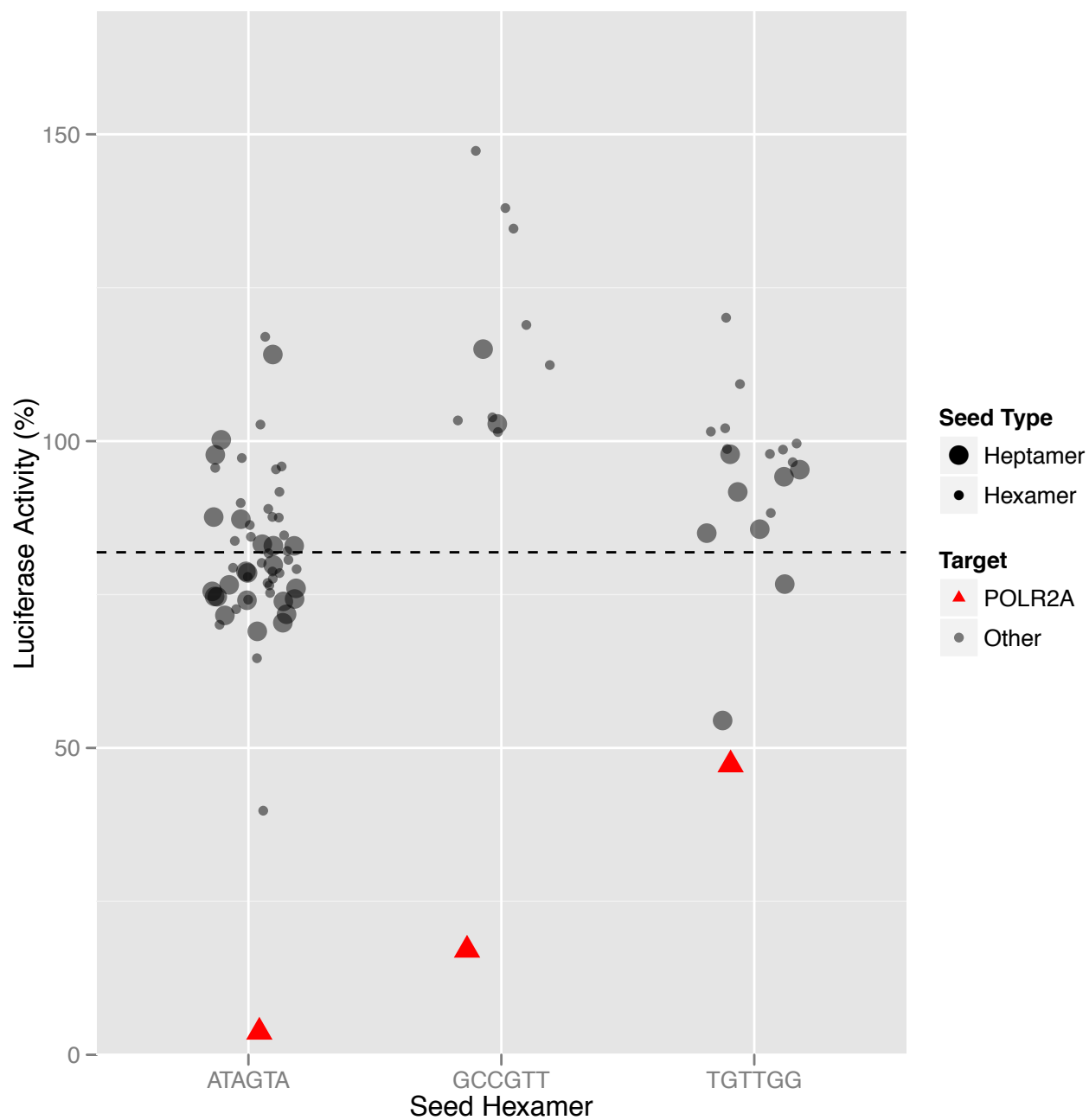

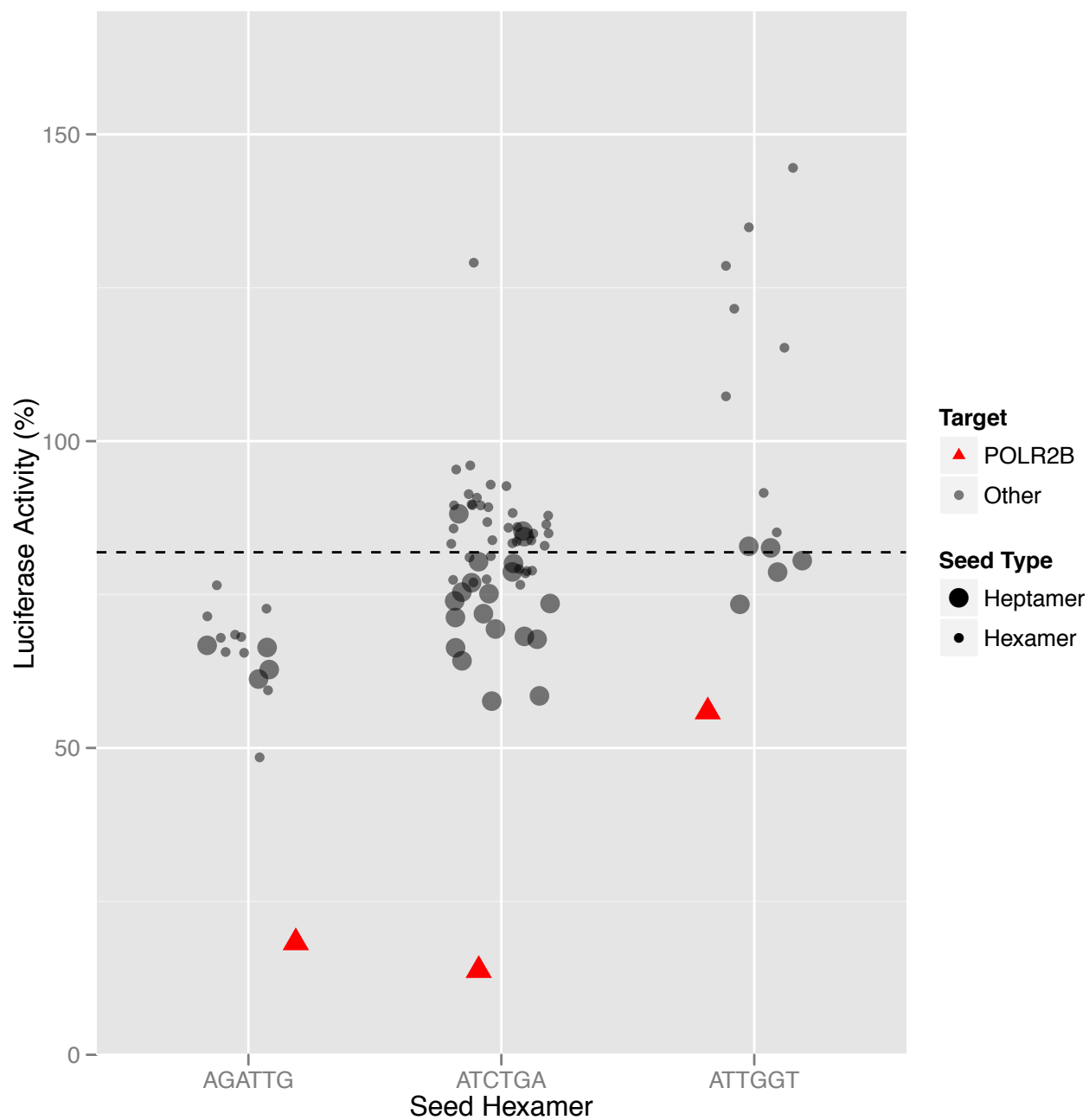

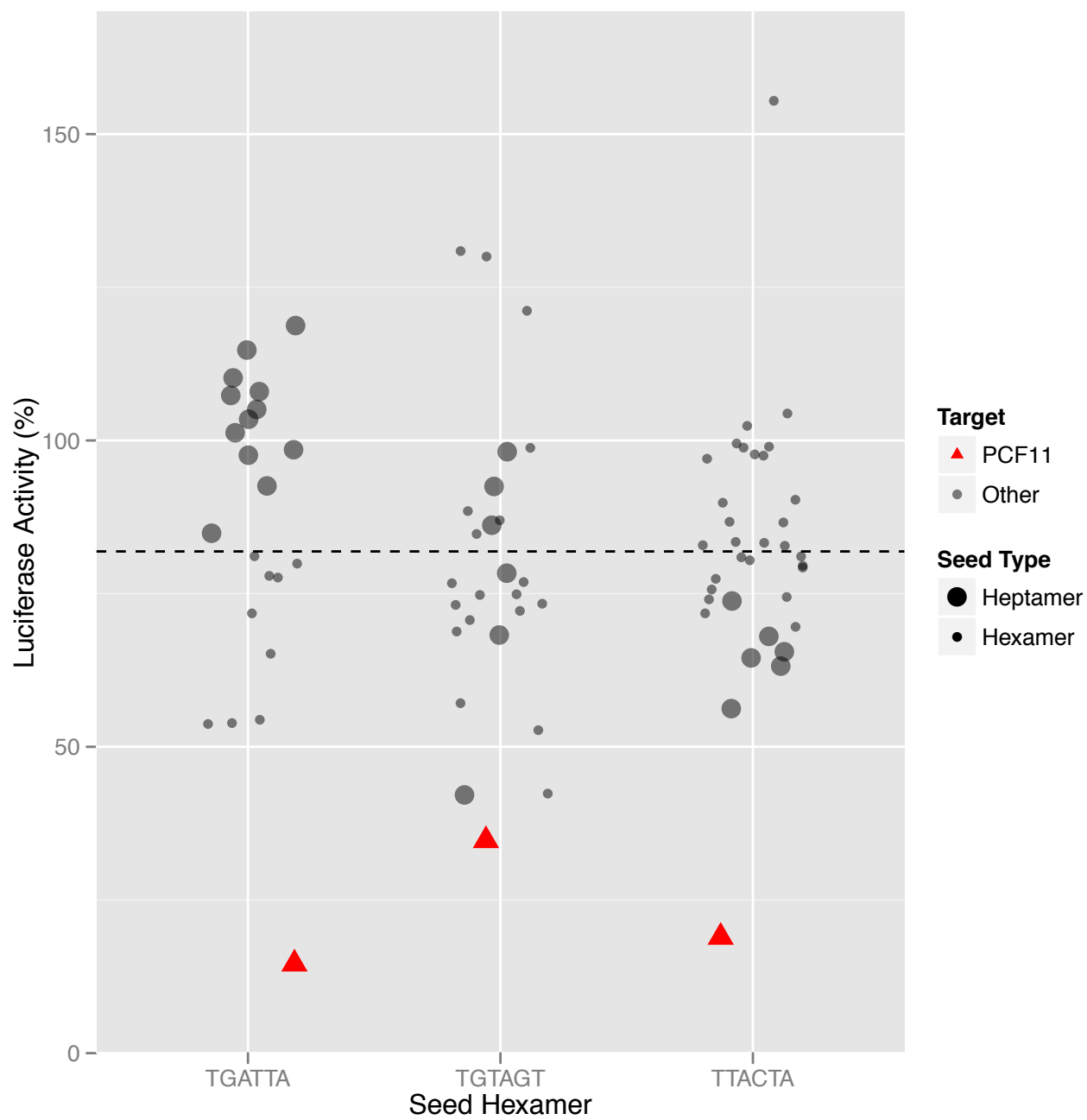

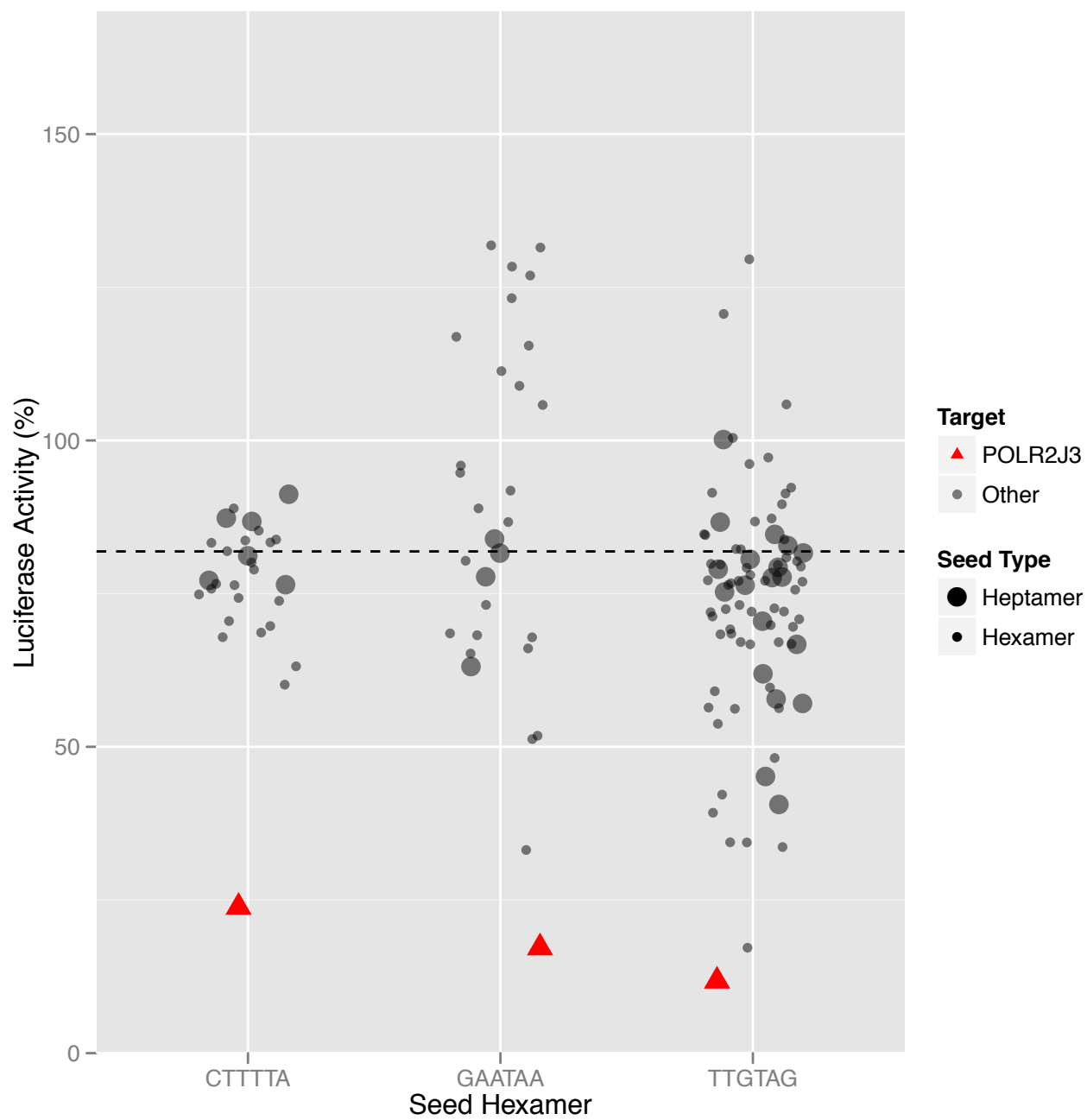

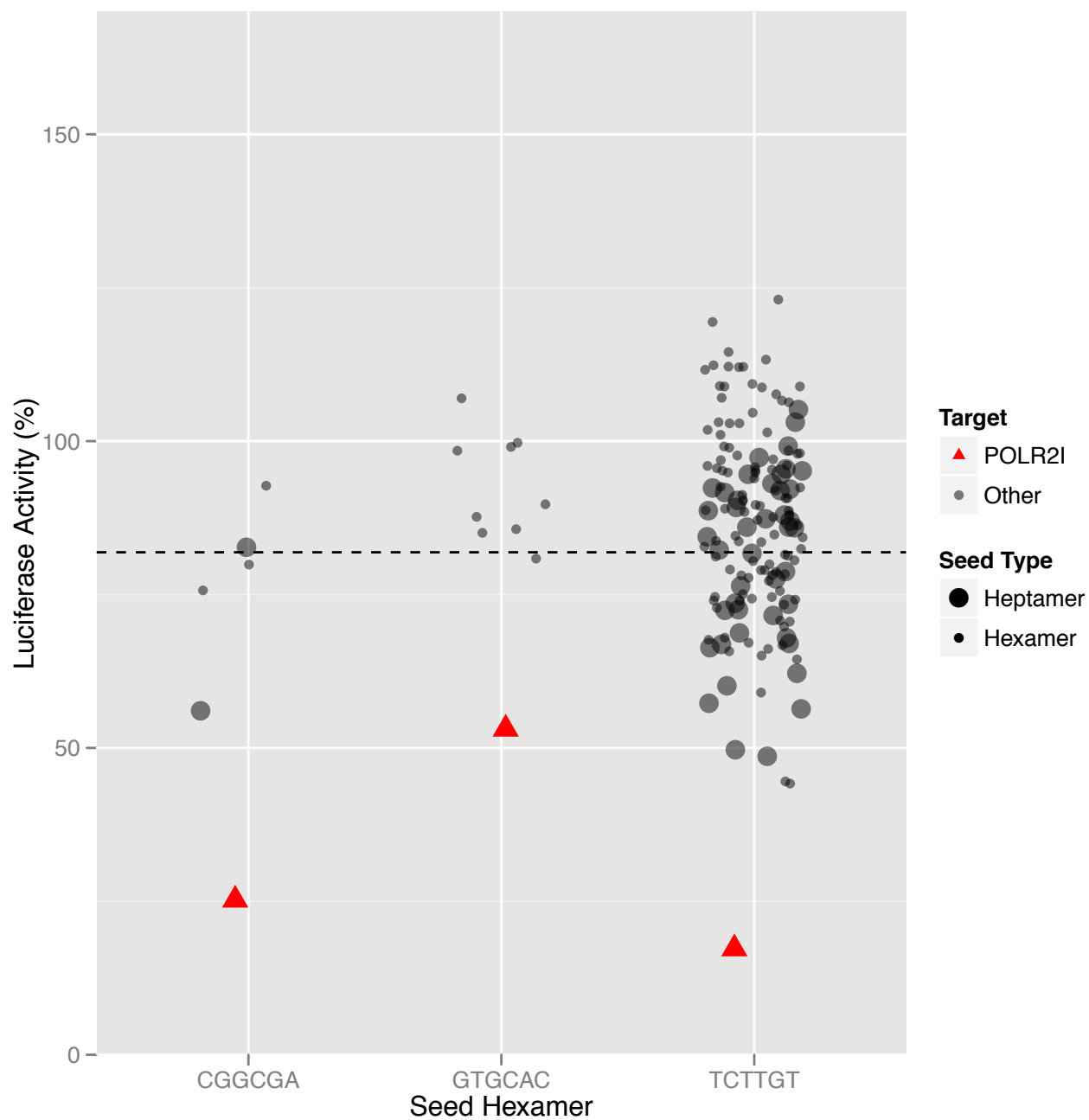

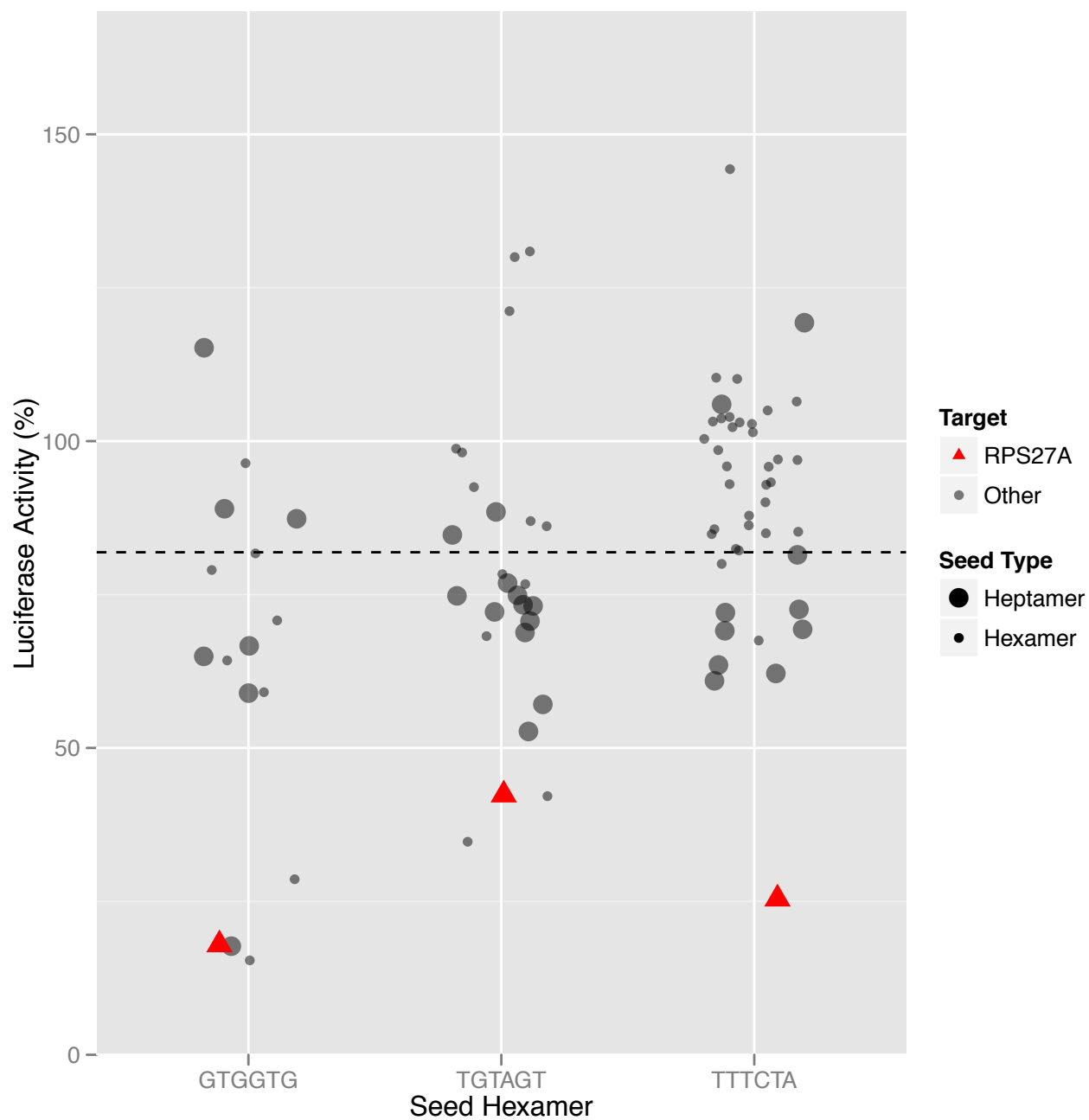

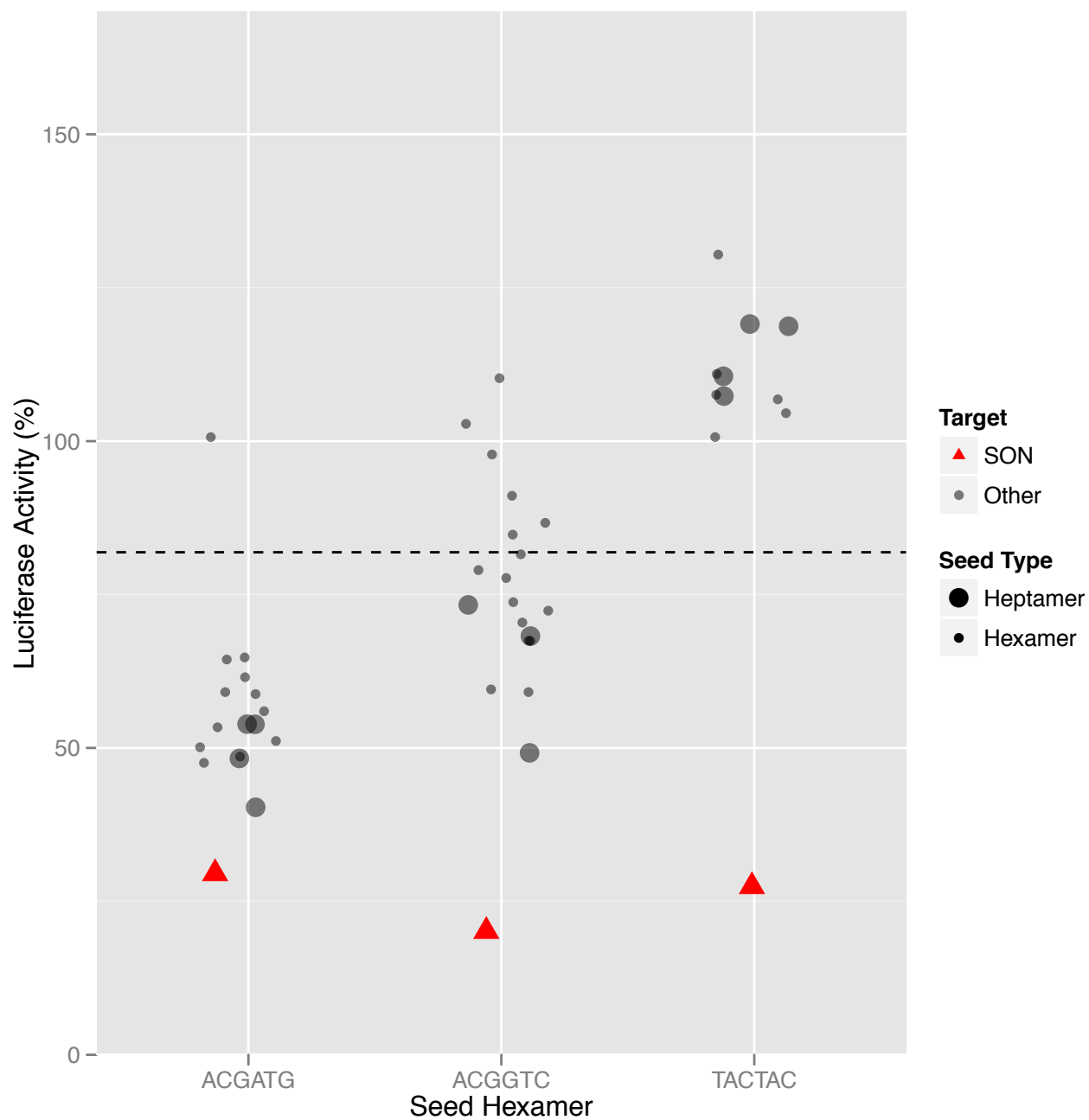

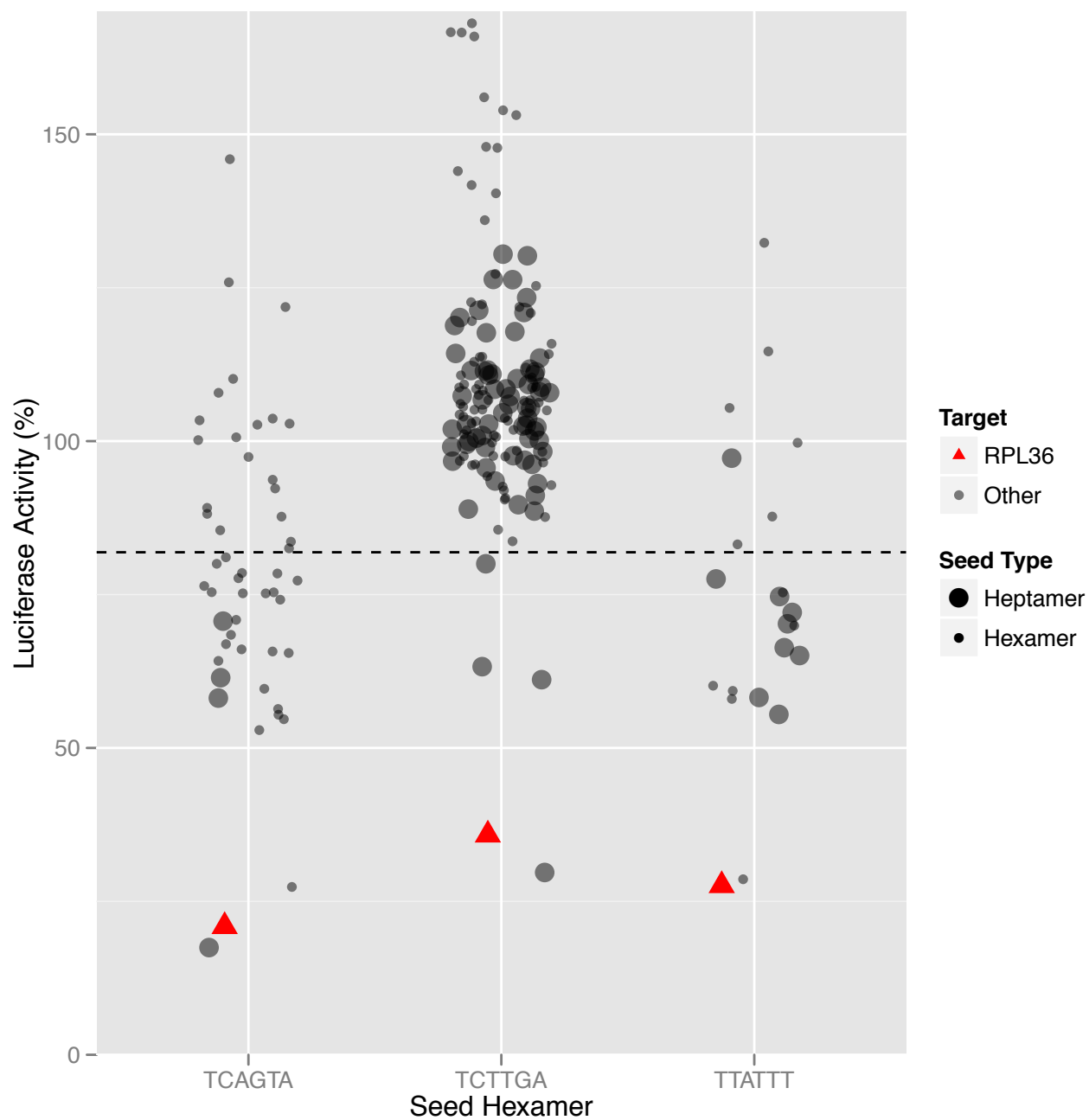

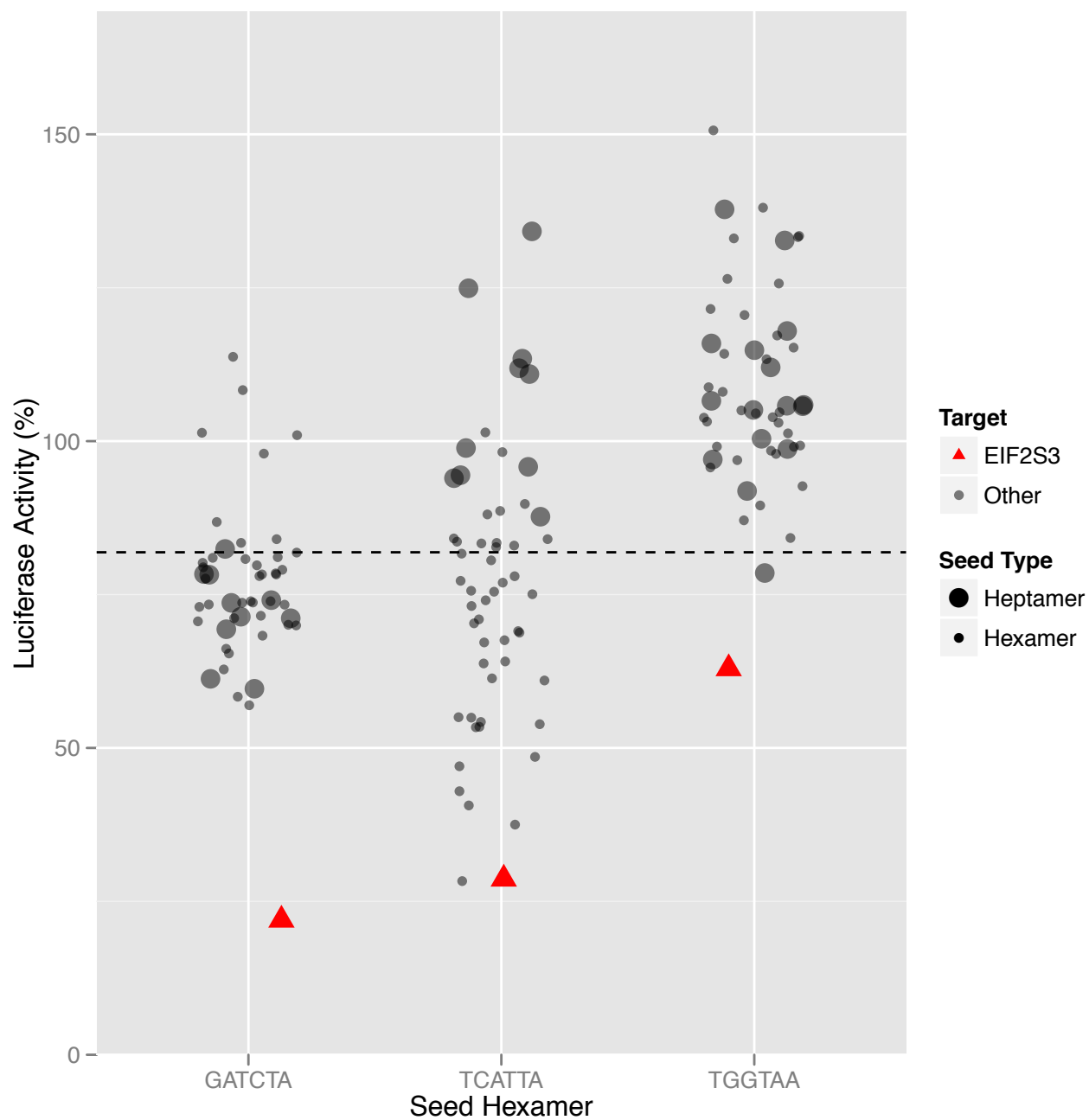

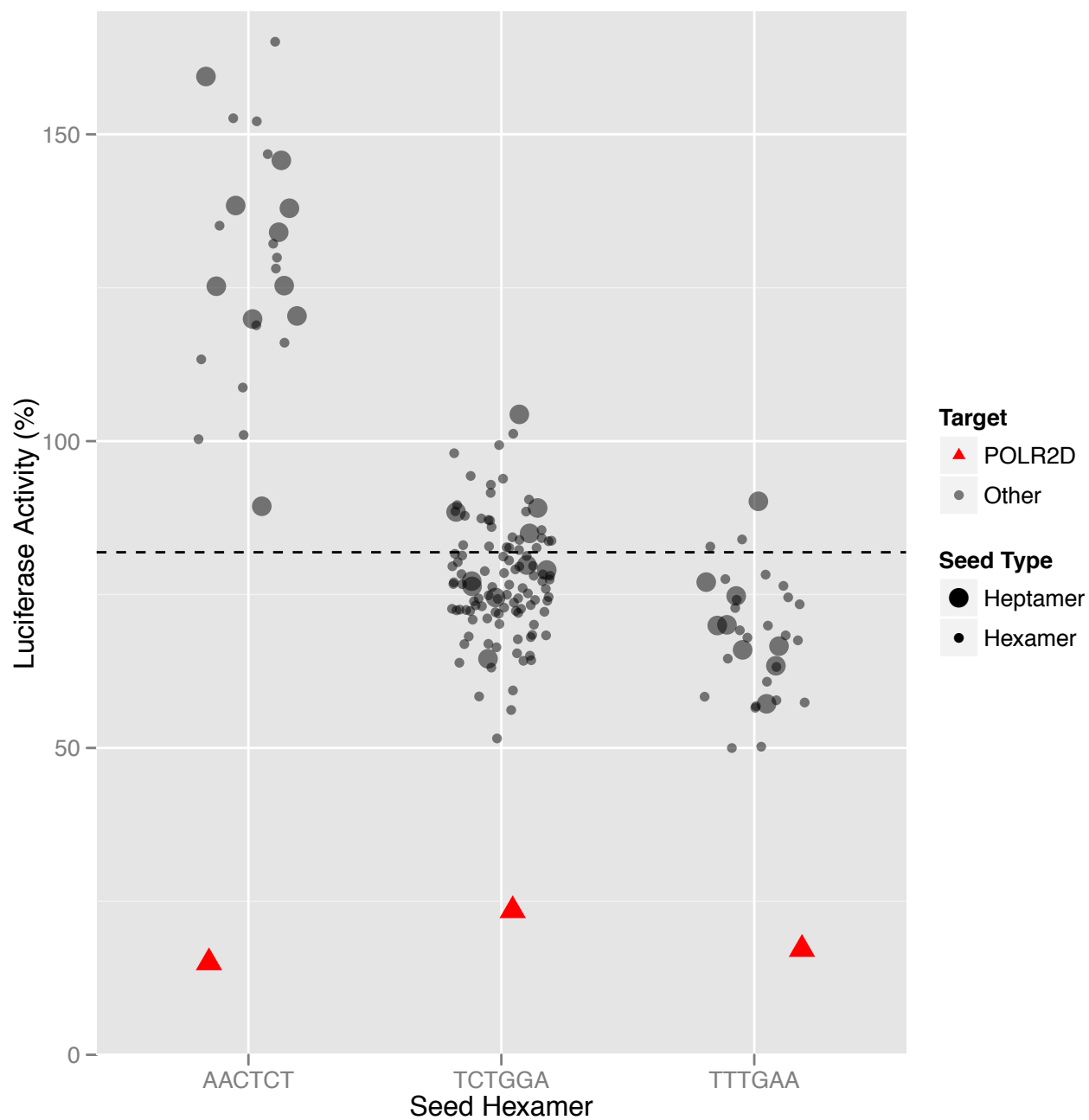

Supplement: Plots S1 — Common Seed Analysis (CSA) Plots for Gold Standard Selections. This supplemental file contains twenty Common Seed Analysis (CSA) plots, one per page. Each CSA plot is for one of 20 siRNAs chosen as a true or false positive, along with the other siRNAs intended to target the same gene. The dashed line represents the median response for the whole genome library. The y-axis is percent luciferase activity compared to negative control. Each siRNA tested against the gene of interest is plotted in its own column as a red triangle. In the same column, siRNAs tested against different genes/mRNAs that had the same heptamer seed sequence (bases 2–8, large grey circles) or hexamer seed sequence (bases 2–7, small grey circles) are plotted. When all siRNAs with the same seed sequence have roughly the same phenotypic effect as the siRNA of interest, we can conclude that the phenotype is likely due to seed-based off-targeting and is not specific to the intended target. (PDF) [file pone.0051942.s002.pdf]
